# Telemedicine and Its Application in Cystic Fibrosis

**DOI:** 10.3390/jpm13071041

**Published:** 2023-06-25

**Authors:** Valentina Fainardi, Gaia Capoferri, Marco Tornesello, Giovanna Pisi, Susanna Esposito

**Affiliations:** Cystic Fibrosis Unit, Pediatric Clinic, Department of Medicine and Surgery, University Hospital of Parma, 43126 Parma, Italy; valentina.fainardi@unipr.it (V.F.); gaia.capoferri@unipr.it (G.C.); marco.tornesello@unipr.it (M.T.); gpisi@ao.pr.it (G.P.)

**Keywords:** telemedicine, teleconsultation, telepediatrics, telemonitoring, televisit

## Abstract

The care of cystic fibrosis (CF) traditionally consists of regular visits to the clinic where a multidisciplinary team can visit the patient, adjust treatments and monitor the disease. During the COVID-19 pandemic when access to hospitals and medical environments was very limited, the role of telemedicine was crucial to keep in touch with patients with chronic diseases such as CF. Increasing evidence demonstrates that electronic health can successfully support healthcare professionals in the management of people with CF. The use of devices connected to digital platforms or smartphones results in a continuous flow of data that can be shared with the clinician and the team in order to improve the knowledge of patients’ diseases and the level of care needed. This narrative review aims to describe the application of telemedicine in CF disease with pros and cons. A literature analysis showed that telemedicine has several advantages in the management of patients with CF. With the evolving support of digital technology, telemedicine can promote clinical visits, adherence to daily treatment, including respiratory physiotherapy and physical exercise, early identification of pulmonary exacerbations and management of psychological issues. The main disadvantages are missed physical exam findings, lack of physical contact that can prevent conversation on sensitive topics, lack of access to technology and lack of technological skills. Furthermore, healthcare operators need appropriate training for telemedicine systems and need time to organise and analyse data generated remotely, which may increase the burden of daily work. Hybrid personalised care models that marge telemedicine and traditional care can be an ideal solution.

## 1. Introduction

Cystic fibrosis (CF) is a monogenic disease considered to affect at least 100,000 people worldwide, mainly Caucasians, with an incidence of 1:3000–4000 individuals [1,2,3]. CF is inherited by the autosomal recessive transmission of a mutation in *CFTR*, a gene located on chromosome 7q31.2, which encodes the cystic fibrosis transmembrane conductance regulator (CFTR) protein. Currently, the identified *CFTR* mutations, which are more than 2000, have been classified into six groups, depending on the defect of the CFTR protein. The most common CF-causing variant in the Caucasian population is F508del (p.Phe508del), which is a class II mutation. Defects in CFTR protein lead to absent or malfunctioning chloride channels in the apical membranes of bronchial or glandular epithelium, resulting in thick and viscous mucus, which is responsible for chronic lung infections, pancreatic and liver dysfunction and reduced fertility [4].

At the time of its first description in 1938, CF was fatal in childhood. Over the last decades, due to early diagnosis with newborn screening, dissemination of evidence-based guidelines to optimise nutritional and pulmonary health, development of CF-specific interdisciplinary care centres and new therapies, the median survival increased up to 52 years in 2018 [1], and now about 50% of people living with CF are 18 years and older [2]. In 2012, the release the innovative *CFTR* modulators in the market resulted in significant improvements in lung function and nutritional status in many patients [5].

The traditional care for people with CF is based on regular visits to the CF clinic, usually every 3–4 months [6]. Visits include (1) clinical assessment with particular attention to respiratory symptoms and signs, growth and nutritional status; (2) measurement of lung function using lung function tests like spirometry and multiple breath washout; (3) microbiological culture on sputum or cough swab; (4) dietary; and (5) respiratory physiotherapy consultation. At least once a year, blood tests and liver scans are performed. These visits require a certain amount of time, which means a working day loss for the parent or the patient, and a school day loss if the patient is a child [6]. In addition, travel and parking can impact the economy of the family, especially for patients living far away from the CF centre.

Telemedicine, also known as “telehealth”, involves the utilisation of electronic devices in order to exchange information from one site to another and to keep patients and their healthcare providers in contact, especially those living in remote geographical areas [7]. The main aim of telemedicine is to improve the patients’ clinical health status [8]. The World Health Organization (WHO) defines telemedicine as the “delivery of health care services where patients and providers are separated by distance. Telehealth uses information and communication technology (ICT) for the exchange of information for the diagnosis and treatment of diseases and injuries, research and evaluation, and for the continuing education of health professionals” [9]. Studies have shown that the activation of a telemedicine system in CF enables the patient to monitor certain biometric parameters, such as oxygen saturation, weight and respiratory function at home, and send them directly to the reference centre [10]. This narrative review aims to describe the application of telemedicine in CF disease with pros and cons. The MEDLINE–PubMed database was searched from 2000 to March 2023 to collect the literature. The following combinations of keywords were used: “telemedicine” AND “cystic fibrosis”. We also performed a manual search of the reference lists of the obtained studies.

## 2. History of Telemedicine and Its New Era during COVID-19 Pandemic

The first application of telemedicine dates back to the XIX century, during the American Civil War, when the telegraph was used to transmit casualty lists and order supplies [11]. After the invention of the telephone, telemedicine assumed and maintained a central role in medical communications. In Italy, the first experiments with telemedicine started in the early 1970s, when the hospital of the Catholic University of Rome set up a teleconsulting service for poisoning, which later became a nationwide system [12]. In 1976, the University of Bologna introduced a prototype system for the acquisition and transmission of electrocardiograms (ECGs) via a telephone network [12]. In the same year, the Research Center of the Public Telecommunications Company (CSELT) established a teleconsulting service between San Giovanni Hospital in Turin and a hospital in Susa, a town that was about 50 km away [12]. This service was used by the emergency department and employed a prototype fax and a video telephone.

In recent years, the rapid development of digital technology has transformed the world of telemedicine. A growing number of apps are being created to involve patients in the care of their disease. Wearable devices and biometric monitors are constantly improving their functions to provide an accurate assessment of vital functions and global access to smartphones, tablets and the Internet potentially allows everyone to get in touch with health systems and operators [10,13]. The remote connection between the patient and the clinician using telecommunication and Internet technology can be used for consultation and intervention. Furthermore, telemedicine can also be used for the purpose of supervision and education (teleducation), consultation with more experienced colleagues (teleconsultation), and research (teleresearch) [8,14,15].

In March 2020, when the emergency committee of WHO declared COVID-19 caused by SARS-CoV-2 a global pandemic, the clinical practice of health professionals profoundly changed in order to respect the new health policies realised to prevent the spreading of the infection. Telemedicine was immediately considered an ideal tool to face this emergency [16], and in the first 4 months of COVID-19 pandemic, the use of telemedicine in the United States (US) increased by 154% [17]. During the lockdown, the need to communicate between specialists located in different hospitals or with patients accelerated the implementation of telemedicine [18]. Publications on “telemedicine and cystic fibrosis” rose from 5 to 10 per year before the pandemic to more than 40 in 2021 [10].

## 3. Telemedicine for Clinical Visits in Cystic Fibrosis (CF)

Telemedicine was previously used in CF patients, but a significant boost in this field was determined by the pandemic [10]. Since CF patients were prevented from accessing hospitals or clinics, digital platforms to communicate with specialists were quickly created and home spirometers were provided [19]. The health professionals based at the CF centre of the Royal Brompton Hospital (UK) reported generally good feedback for telemedicine. With telemedicine, visits were more on time, more frequent and performed when needed by the patient. Furthermore, home-based visits made the patient feel safer [19]. The additional benefit of chest auscultation has been reported as relatively minor, particularly in stable patients. However, digital platforms and home spirometers are available in a few countries, and validation of these devices should be ensured in order to capture reliable data.

Wearable devices like fitness trackers and accelerometers capable of measuring steps, heart rate, oximetry or respiratory rate, and apps installed on smartphones can produce a continuous flow of data that can describe and monitor the disease in terms of oxygen saturation and cardiac and respiratory rate [10]. The use of home devices, like a spirometer or blood glucose monitor, can further provide the patient with useful information to assess his condition and adjust the treatment [10]. All this can be shared with the specialist immediately or at set intervals via digital technology (emails, digital platforms and apps).

In a study protocol developed to increase adherence in CF patients, five cardinal points have been identified to guarantee successful home monitoring and management of patients with CF with telemedicine: telephone coaching by instructed staff, video conferencing between the patient and the medical personnel, apps and data servers to check adherence [20]. Home monitoring aims to (1) support adherence to daily treatments; (2) facilitate the early identification of pulmonary exacerbations; (3) increase engagement in respiratory physiotherapy and physical exercise; and (4) offer psychological support.

**Figure 1:**
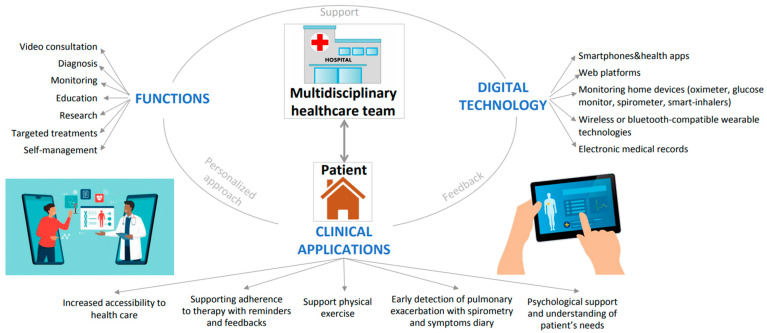
Telemedicine in cystic fibrosis (CF) patients: a close relationship between multidisciplinary team and patient despite the distance. On the right are the devices that can be used to communicate the patient’s data and needs to healthcare operators; on the left are the several functions that telemedicine can offer the clinician and the patient; and on the bottom are the clinical applications that can be used in telemedicine for the management of CF. Support, feedback and personalised approaches are crucial characteristics of telemedicine.

Figure 1 describes the devices that can be used by the multidisciplinary team to monitor the patient and summarizes the clinical applications and the functions of telemedicine in CF care.

### 3.1. Supporting Adherence to Daily Therapy

According to the Cystic Fibrosis Trust report published in 2018, children with CF in the United Kingdom (UK) spend around 137 min/day on treatment [21]. This significant treatment-related burden leads to frequent non-adherence to therapies, which has been identified as the leading cause of treatment failure [21]. The promotion of adherence to therapies is one of the greatest challenges for healthcare providers. Therefore, assessing adherence is crucial for the clinician to understand whether the deterioration in the clinical condition is due to the progression of the disease or insufficient treatment [6].

Adherence can be investigated using self-reporting methods (usually considered poorly accurate) or computerised methods [22,23,24,25,26]. The latter includes digital electronic adherence recordings and data-tracking nebulizers. Digital electronic adherence recordings like medication possession ratio (MPR) and prescription refill data were found to increase patient compliance, particularly in adolescents [22,23]. Data-tracking nebulizers are a more advanced option for asthma patients to monitor not only if the patients are taking their medication but also their inhalation techniques [24]. In 2020, Drabble et al. described the findings of a web-based adherence monitoring system with health professional support to help adults with CF increase their adherence to nebulizer treatments [25]. Real-time data on adherence obtained with a nebulizer with electronic monitoring capabilities were visible to the patients and health professionals delivering the intervention. Each intervention was personalised depending on the motivation of the patient and included feedback and personalised plans to increase knowledge and education, empower patients and support behavioural change. Visibility to both the self and professional operators was an important motivator to improve adherence. Interestingly, building a relationship with a doctor was associated with a feeling of being accountable for someone and, therefore, with increased adherence [25]. In a recent Cochrane review that included this latter study, it was concluded that compared to usual care, digital intervention plus tailored support via an online platform may improve adherence to inhaled therapy, which is likely to lead to a slight reduction in treatment burden in the medium term (low- and moderate-certainty evidence) [26]. However, further studies are needed to demonstrate their influence on the quality of life. Moreover, inhalation techniques (e.g., correct body position and correct flow during inspiration) cannot be monitored yet.

In a post hoc exploratory analysis of the ACtiF trial, a large UK multicentre study, adherence was extrapolated from data-logging nebulisers in 543 adult patients [27]. The results demonstrated that patients poorly adherent (adherence < 50%) to inhaled antibiotics had a higher FEV1 variability than patients who were moderately (50 to <80%) and highly adherent (≥80%), with median values of 8.1%, 6.3% and 6.3%, respectively [27].

Respiratory physiotherapy represents a substantial portion of the daily care for CF patients. Although perceived as very important, airway clearance techniques (ACTs) such as the positive expiratory pressure (PEP) technique are considered the most burdensome daily treatment [28]. The so-called “serious gaming” is a promising option to increase adherence to treatment with playing [29]. Breath pressure sensors were inserted into the ACT device in order to make daily physiotherapy more fun. Transforming respiratory therapy into a digital game may reveal promising results in increasing adherence; however, at present, evidence is scant. Project Fizzyo is an example of an ongoing trial with a bespoke breath pressure sensor inserted into the ACT device and an activity tracker that records physiotherapy sessions and stimulates adherence via gaming [30].

A further part of the daily treatment for most patients with CF is the supplementation with pancreatic enzymes. An app called MyCyFAPP, created to optimise nutritional status in children with CF, was used in a cohort of 171 children aged 2 to 17 years from six European CF centres [31]. This app consisted of a diary where patients could record meals and then be advised of the appropriate amount of enzymes, a symptom diary where patients report gastrointestinal symptoms, educational material and a link to the professional digital tool containing follow-up charts showing the evolution of nutrient intake and symptoms over time and enabling direct communication between patients and healthcare professionals. After 6 months, the quality of life and abdominal symptoms improved significantly, and the same results were perceived by their parents [31].

### 3.2. Supporting Physical Exercise

Although a recent Cochrane review concluded that current evidence shows little or no effect of physical exercise on lung function and quality of life [32], it is recommended that all children practice at least 60 min of physical activity every day [33]. Adults should take part in at least 150 min of moderate vigorous-intensity aerobic physical activity or 75 min of vigorous-intensity physical activity per week [33]. There are no specific guidelines for physical activity in CF, but regular physical exercise is considered a fundamental part of CF treatment care. Physical exercise enhances sputum clearance, strengthens all muscles, including those involved in respiration, ameliorates lung function and promotes improvement in the quality of life [34,35,36]. Spending at least 30 min per day practicing regular physical activity of moderate intensity is associated with better peak oxygen uptake (V’O2 peak), a prognostic factor for CF evolution [37,38]. However, the adherence rates to physical exercise are variable [39].

The most validated tools that measure energy expenditure are accelerometers like SenseWear Pro3 Armband (SWA), which, however, are very expensive and usually used only for research purposes [40]. Savi and colleagues in a research study compared the use of SWA with everyday use technologies, such as Smartwatch, Fitbit, Android and iOS smartphones, to monitor physical activity in adult CF patients [41]. Interestingly, Fitbit and iOS smartphones were comparable to SWA in step counting, and Fitbit was also comparable in terms of energy expenditure, suggesting a promising role of these devices in daily practice [41].

A systematic review exploring telemedicine-based exercise in patients with CF included eight studies performed in children and adults (n = 180) with different programs lasting on average 12 weeks involving video or telephone calls [42]. Significant improvements were found in the Cystic Fibrosis Questionnaire—Revised (CFQ-R) respiratory domain in most studies, indicating a beneficial impact on the perceived quality of life; no significant benefits were reported on FEV1 [42]. A home-based resistance exercise training (RET) programme in adolescents with CF and impaired glucose tolerance using virtual personal training suggested a positive impact on insulin secretion, body composition and exercise capacity [43].

Considering the high prevalence of gaming technology, incorporating exercise into gaming may be a strategy to help people engage in self-monitoring exercises. A randomised controlled trial including 39 children with CF compared the effect of a home-based active video game programme on exercise capacity, muscular strength and quality of life using the CFQ-R with routine care [44]. The experimental group (n = 19) started a 6-week home training protocol with daily exercise (30–60 min of running, squats, lunges and bicep curls) using Nintendo Wii™ 5 days a week. The game was supervised by a virtual personal trainer and included a heart rate (HR) monitor. To increase patient adherence, a physiotherapist conducted weekly telephone check-ins. After the training period, patients were instructed to continue their individualised exercise programme using the same programme at home for a 12-month follow-up period, with an exercise prescription of a minimum of 2 days per week, 20 min per session. The results showed short- and long-term training effects in the experimental group (improved muscle performance and quality of life), but long-term adherence to the home programme progressively decreased, highlighting the need for continuous support to promote patients’ regular engagement [44]. When a training intervention is integrated into the home environment and uses personalised exercises with all sessions supervised by an exercise therapist with frequent and consistent encouragement, patients’ satisfaction and compliance are reported to be high [43,45]. From preliminary data, what seems promising is the use of social media and web-based platforms for online exercise classes with instructors who have CF [24].

### 3.3. Early Detection of Pulmonary Exacerbations

Regular daily treatment aims to decrease exacerbations associated with progressive decline in lung function, hospital admissions and increased mortality rate [46,47,48,49,50]. Early detection of exacerbations is one of the goals of CF care. Up to 25% of patients experiencing an exacerbation fail to recover their initial lung function [51]. Delayed reporting of worsening symptoms results in delayed initiation of treatment, which has been shown to be associated with failure to recover to baseline [51].

The combination of lung function, vital signs, like heart rate and oxygen saturation, and respiratory symptom scoring can predict an exacerbation [50]. These data can be shared with the multidisciplinary team of the CF centre via different modalities (e.g., Wi-Fi, Bluetooth and email) so that the CF centre can contact the patient to start adequate treatment. Smartphone applications can be successfully used to report symptoms and communicate with clinicians to decide on the need for antibiotic treatment [52], and wearable devices that record respiratory rates may promptly suggest the onset of an exacerbation [53]. In a 6-month prospective study, a real-time electronic monitoring system associated with an electronic diary in which the patient could record symptoms and spirometry once daily had a poor uptake among CF patients, although for those who completed the study, this method detected pulmonary exacerbations early [54]. In a multicentre, randomised trial performed in 14 CF centres, children in the intervention group measured lung function at home and symptoms electronically twice per week, while the control group was seen every 3 months [55]. No significant difference between the study arms in FEV1 was seen in 52 weeks. However, exacerbations were detected more frequently in the intervention arm. Similar results were reported in a recent Australian clinical trial on 60 young adults with CF using a smartphone application with yes/no questions relating to symptoms suggestive of a respiratory exacerbation [56]. Participants in the intervention group were given the possibility to contact the CF centre in case of worsening symptoms rather than presenting to the scheduled follow-up control. Despite the same number of exacerbations over the 12-month follow-up, the number of courses of oral antibiotics increased, and the median time to the detection of exacerbation requiring oral or IV antibiotics was shorter. The authors described that the median time to the detection of exacerbation requiring oral or intravenous antibiotics was shorter in the intervention group compared to the control group [56]. Notably, in several studies, the effectiveness of telemedicine in detecting exacerbations has been compromised by suboptimal compliance with the intervention [55,57,58,59]. Furthermore, telemedicine failed so far to show an improvement in lung function.

### 3.4. Psychological Support

Different studies have shown that people with chronic conditions and their caregivers are at a higher risk of depression and anxiety [60]. These psychological symptoms in patients with CF may result in decreased adherence to therapy, more hospitalisations and earlier mortality [61]. Hence, the Cystic Fibrosis Foundation (CFF) and the European Cystic Fibrosis Society developed guidelines for mental health in CF encouraging annual screening for depression and anxiety in patients and caregivers [62]. Due to the easy interaction with healthcare professionals, telemedicine may play a crucial role in supporting CF patients with psychological issues.

During the COVID-19 pandemic, the CF team of Bambino Gesù Children Hospital (Rome, Italy) developed a Telehealth Psychological Support Intervention for adolescents and young adults with CF, as well as their caregivers, providing them with cognitive-behavioural strategies to reduce stress and emotional challenges during lockdown [63]. Participants (patients aged 12–36 years and caregivers of patients younger than 18) completed four individual telehealth sessions with a psychologist focused on self-care, coping skills, exercises to improve mood and individual emotional challenges and using screening tools, such as the Patient Health Questionnaire-8 item (PHQ-8) and Generalized Anxiety Disorder-7 item (GAD-7). This intervention showed that stress decreased from pre- to post-testing in both patients and parents. Both groups reported a high rate of depression and anxiety at baseline (71%). Depression, but not anxiety, significantly decreased after intervention (38%) [63].

During the COVID-19 pandemic, the frequency of mental health screening decreased in 60% of American CF centres [64]. Alternative methods for in-person visits, like videoconferencing, telephone or mail were used. A cross-sectional survey-based study conducted in 11 CF centres in the US was performed via telehealth visits between April 2020 and June 2020, reporting a high rate of satisfaction in both adult patients and children’s caregivers [65]. The main reason for this was the adequate amount of time spent by the team addressing the patient’s concerns and questions. The commonest concern reported was the lack of routine tests performed in CF clinics, like throat/sputum cultures and pulmonary function testing. Despite this, most patients (53% adults and 69% children) expressed an interest in telehealth as part of future care [65]. A survey conducted in our CF Unit (Parma, Italy) revealed that patients still preferred traditional in-person visits, but most of them were keen to use telemedicine and home spirometry and share data with the medical team (unpublished data). The main concern was the lack of physical examination and, therefore, the possible inaccuracy of the video visit compared to the in-person visit (personal communication).

## 4. Conclusions

Telemedicine has several advantages in the management of patients with chronic diseases like CF. With the evolving support of digital technology, telemedicine can promote clinical visits, adherence to daily treatment, including respiratory physiotherapy and physical exercise, early identification of pulmonary exacerbations and management of psychological issues [10]. The main disadvantages are missed physical exam findings and lack of physical contact that can prevent conversations on sensitive topics. However, the possibility of frequent contact between the patient and the multidisciplinary team can increase the interaction and knowledge of the clinical status of the patient, resulting in close monitoring of the disease. Moreover, the lack of microbiological surveillance could be considered a disadvantage because in the case of pathogens such as *Pseudomnas aeruginosa*, early detection increases the probability of successful eradication. Home collection of sputum samples is feasible but requires appropriate infrastructure [66]. Another disadvantage is that the differences between patients with digital skills and those without them are increasing, which especially affects older patients and patients with insufficient Internet access [10]. Other possible limitations are the lack of access to technology, like Internet access or devices, and the lack of technological skills. Furthermore, healthcare operators need appropriate training for telemedicine systems and need time to organise and analyse data generated remotely, which may increase the burden of daily work. Table 1 summarizes the advantages and disadvantages of telemedicine in CF.

Telemedicine is not for all and needs to be tailored to the specific needs of the patient. Hybrid personalised care models that merge telemedicine and traditional care can be an ideal solution. Telemedicine can be particularly useful in the era of *CFTR* modulators since patients will live longer, and more patients will work and have families, which means having less time to dedicate to in-person visits. Furthermore, patients on modulators may have less need for visits due to their improved physical condition and may show decreased adherence to other treatments since many discontinue their therapies.

## Figures and Tables

**Table 1:** Advantages and disadvantages of telemedicine in cystic fibrosis (CF).

Advantages	Disadvantages
Convenience in clinical visits, especially for those living far away or those with transportation issues	Technical difficulties
Easier and quicker access to care	Need for digital charts to access patients’ data
Lower infection risks; contact when needed	Lack of microbiological surveillance
More knowledge of the home environment	Digital divide
Increased adherence to treatments	Need for appropriate training both for health operators and patients
Support and supervision of physical exercise	High costs
Comfortable environment	Long-term adherence is sometimes low
On-time consultations	Lack of physical examination
	Lack of face-to-face interaction

## Data Availability

Not applicable.
